# Trends of T2 and Non-T2 Cytokines During Mepolizumab Treatment in Different Asthma Patient Subgroups

**DOI:** 10.3390/biomedicines14030541

**Published:** 2026-02-27

**Authors:** Marco Zurlo, Francesca Ambrosani, Matteo Maule, Naila Arif Cheema, Francesca Mascia, Antonino Aparo, Giuseppe Argentino, Rachele Vaia, Anna Baiocchi, Gianenrico Senna, Simonetta Friso, Annalisa Castagna, Marco Caminati

**Affiliations:** 1 Department of Medicine, University of Verona, 37134 Verona, Italy; marco.zurlo1@gmail.com (M.Z.); francesca.ambrosani@univr.it (F.A.); matteo.maule@univr.it (M.M.); nailaarif.cheema@univr.it (N.A.C.); francesca.mascia94@gmail.com (F.M.); giuseppe.argentino@univr.it (G.A.); rachele.vaia@univr.it (R.V.); anna.baiocchi@studenti.univr.it (A.B.); gianenrico.senna@univr.it (G.S.); simonetta.friso@univr.it (S.F.); annalisa.castagna@univr.it (A.C.); 2 Allergy Unit and Asthma Center, Integrated University Hospital of Verona, 37100 Verona, Italy; 3 Research Center LURM (Interdepartmental Laboratory of Medical Research), University of Verona, 37134 Verona, Italy; antonino.aparo@univr.it

**Keywords:** cytokines, asthma, EGPA, CRwNP, mepolizumab

## Abstract

**Background:** The anti-IL-5 monoclonal antibody, mepolizumab, has shown clinical efficacy and safety for the treatment of severe eosinophilic asthma (SEA), chronic rhinosinusitis with nasal polyps (CRwNP) and eosinophilic granulomatosis with polyangiitis (EGPA). We aimed to investigate the trajectories of the inflammatory cytokines at the systemic level during mepolizumab treatment, in SEA, SEA with CRwNP, and EGPA. **Material and Methods:** Treatment response was explored within a real-life observational prospective study. Clinical, functional and inflammatory outcomes as well as serum T2 (IL-4, IL-5 and IL-13) and non-T2 cytokine trends (including IL-5, IL-6, IL-13, IL-10) were evaluated at baseline and 6–12 months after mepolizumab initiation. **Results:** Overall, 45 patients were consecutively enrolled (SEA: 18; SEA with CRwNP: 9; EGPA: 18), including 27 females, with an average cohort age 60.65 years. Clinical parameters (FEV1, FeNO, SNOT 22, ACT, blood eosinophil count) improved in the different subgroups regardless of coexisting determinants of potential higher disease burden, including CRwNP and previous EGPA history. Cytokine analysis revealed heterogeneous profiles at baseline and statistically significant changes in IL-5 and IL-10 concentrations within the same disease subgroup at different time points. **Conclusions:** Our observations suggest the ability of mepolizumab to modulate both T2 and non-T2 immune pathways and highlight the persistence of slightly different molecular profile in different severe asthma patients depending on concomitant conditions, which is relevant for the long-term follow-up and potential association therapy combining options which address different targets. More research and larger studies are needed.

## 1. Introduction

Asthma is a common respiratory disease characterized by wheezing, shortness of breath, chest tightness and coughing. It is the second leading cause of mortality among chronic respiratory illnesses. According to the Global Asthma Report, the global prevalence of asthma is 9.1% among children, 11.0% among adolescents, and 6.6% among adults [1].

Severe asthma accounts for 5–10% of asthma patients [2,3], with many determinants contributing to its burden [3], including coexisting chronic rhinosinusitis with nasal polyps (CRwNP) or a previous history of eosinophilic granulomatosis with polyangiitis (EGPA).

CRwNP represents a common comorbidity in severe asthma patients, affecting at least half of them and significantly increasing the overall disease burden [4,5]. Individuals affected by both conditions usually report more severe sinonasal symptoms, more severe respiratory symptoms and more inflammatory involvement. Therefore, these patients experience a higher impact of disease on their health-related quality of life [6].

In fact, individuals suffering from both conditions experience more severe sinonasal symptoms, more extensive inflammation in the lower airways, more compromised pulmonary function, and a decreased health-related quality of life (HRQoL) when compared to individuals with asthma or CRwNP only [5,6].

EGPA is a rare systemic necrotizing vasculitis that affects small-to-medium-sized blood vessels. Lung involvement commonly appears as asthma, lung infiltrates, or nodules [7,8,9], while ear, nose the throat (ENT) disease effects includes nasal polyposis and chronic rhinosinusitis [10,11]. Asthma and CRwNP often persist in EGPA patients even when the remission of systemic disease activity has been achieved [12].

Mepolizumab, a monoclonal antibody selectively targeting IL-5, is currently approved for severe eosinophilic asthma and CRwNP at a dose of 100 mg every four weeks and for EGPA at a dose of 300 mg every four weeks [13]. There are increasing amounts of real-life evidence to support the use of low dose mepolizumab in EGPA patients who are in remission phase and for patients with persisting severe eosinophilic asthma [12,14,15].

The pivotal role that IL-5 and eosinophilic inflammation play in the pathophysiology of the three conditions mentioned above represents the immunological rationale for the multiple indications of mepolizumab. However, the clinical expression and the potential disease burden in the three clinical subgroups vary significantly, as do the immune cytokines that interact with IL-5 [16,17,18].

Our real-life observational study aimed to evaluate the baseline molecular profile of severe eosinophilic asthma patients with/without CRwNP or with a previous history of EGPA that had been treated with four weekly doses of mepolizumab 100 mg for 12 months, including the assessment of typical T2 (IL-5, IL-13) and non-T2 (IL-6, IL-10) cytokines. The trends of the same cytokines were prospectively analyzed during one-year mepolizumab treatment at different time points and correlated to clinical/biochemical parameters.

## 2. Materials and Methods

### 2.1. Study Population

Severe eosinophilic asthma patients consecutively referring to our clinic between February 2021 and March 2024, eligible to mepolizumab 100 mg/4 weeks for asthma indication according to the current regulatory requirements [13] and consenting to study participation were enrolled and followed-up for one year. The study was approved by our Institutional Ethic Committee (CESC 2987). At baseline the subjects were allocated to one of the following subgroups according to their profile: (1) severe eosinophilic asthma (label SEA, group A), diagnosed based on ERS/ATS definition [19]; (2) severe asthma with CRwNP (label “SEA with CRwNP”, group B) diagnosed according to EUFOREA definition [20]; (3) persisting severe eosinophilic asthma with a history of EGPA in remission phase at the time of enrolment (label “EGPA”, group C), diagnosed in accordance with ACR/EULAR criteria [21].

Exclusion criteria included unresolved differential diagnoses (e.g., vocal cord dysfunction, obstructive sleep apnea, hyperventilation syndrome, allergic bronchopulmonary aspergillosis, fungal asthma, Carrington disease, or asthma–COPD overlap). Patients were also excluded if they had received prior biologic therapy other than mepolizumab, demonstrated non-adherence to inhaled treatment, were active smokers or former smokers with >20 pack-year history, were on a regular steroid daily dose >10 mg prednisone (or equivalent). Additional exclusions applied to those already enrolled in interventional research, pregnant women, individuals with active malignancy or malignancy in remission <5 years, active parasitic infection or infection within the past 24 weeks, and patients with psychiatric conditions impairing comprehension, protocol adherence, or ability to provide informed consent.

### 2.2. Data Collection

Clinical evaluation was performed and experimental samples (blood sample) were collected for enrolled patients at the time of inclusion and after 6 and 12 months from the start of mepolizumab treatment.

The following clinical variables were considered and recorded: age, sex, body mass index (BMI), Asthma Control Test (ACT), Sinonasal Outcome Test-22 (SNOT-22) in patients with concomitant CRwNP, FEV1 and FEV1% of predicted, fractional exhaled nitric oxide (FeNO), blood eosinophils (eos/μL), and oral steroid treatment (OCS).

Serum samples were analyzed for the presence of the cytokines IL-5, IL-6, IL-10, IL-13 using Simple Plex assays on the ELLA microfluidic immunoassay system (Protein Simple, San Jose, CA, USA). Cytokines selection reflects their known relevance as immunological drivers in the pathobiology of the conditions characterizing the study population. IL-5 and IL-13 orchestrate T2 and eosinophilic inflammation; IL-6 is a major player in non-T2 inflammatory processes, regardless of their specific origin or triggers; IL-10 is a predominant mediator down-modulating inflammatory response and preventing consequent tissue damage [22,23].

Before the analysis, samples were diluted 1:1 with the sample diluent, and 50 µL of the prepared mixture was loaded into each sample inlet of the Ella™ Automated Immunoassay System and Simple Plex™ cartridges (ProteinSimple, a Bio-Techne brand, San Jose, CA, USA) following the manufacturer’s guidelines. The appropriate wells on the cartridge were filled with a wash buffer, and data acquisition was performed using Simple Plex Runner v.3.7.2.0 (ProteinSimple, a Bio-Techne brand, San Jose, CA, USA). Results were generated approximately 90 min after the assay initiation.

### 2.3. Statistical Analysis

Data are presented as mean and standard deviation (SD) for variables with a normal distribution, as median and range for variables with a non-normal distribution, and as percentage for categorical variables. Statistical analyses were performed using R 4.5.2 software. Cytokine levels were compared across diagnostic subgroups and follow-up time points using the Kruskal–Wallis test followed by Dunn’s post hoc test. Cytokines with more than 50% missing values or fewer than five available measurements were excluded from the analysis. Associations between cytokines and clinical variables were assessed using Spearman’s rank correlation coefficient, computed separately within each diagnostic group and follow-up time point. Correlation analyses were performed only when at least four patients with complete paired observations were available. A two-sided *p*-value < 0.05 was considered statistically significant. Significant comparisons are indicated by asterisks as follows: * for *p* ≤ 0.05, ** for *p* ≤ 0.01, *** for *p* ≤ 0.001, **** for *p* ≤ 0.0001.

## 3. Results

Overall, 45 patients were enrolled, including 27 females and 18 males, with an average age of 60.65 years. Table 1 summarizes the baseline clinical characteristics of the study population.

When compared to the other subgroups, EGPA patients presented a better asthma control (ACT and FEV1), despite showing the greatest proportion of continuous OCS use. In terms of inflammation biomarkers, blood eosinophils are higher in SEA and EGPA patients, while the worst FeNO values were detected in SEA with CRwNP patients and EGPA subgroup. Nasal control (SNOT 22 score) was less compromised in EGPA relative to SEA with CRwNP patients.

Table 2, Table 3 and Table 4 illustrate the trends of the clinical parameters across the time points by population subgroups.

In Table 2, a significant improvement of all parameters in the SEA subgroup could be observed. The ACT scores progressively increased, indicating better asthma control. Eosinophil counts significantly dropped over time, consistent with the reduced airway inflammation. Spirometry values improved, indicating better lung function. FeNO levels also showed a slight improvement. Lastly, exacerbation frequency decreased, as indicated by the reduced OCS cycles and the lower portion of patients using continuous systemic corticosteroid treatment, underscoring the therapeutic efficacy of mepolizumab in managing severe asthma over the treatment period.

When compared to SEA subgroup, SEA with CRwNP patients demonstrated similar outcomes over the treatment course, characterized by an overall improvement related to all evaluated parameters. As shown in Table 3, ACT scores exhibited an upward trend, reflecting amelioration in asthma control and symptoms management. Similarly, SNOT22 scores showed a marked reduction, indicating better sinonasal health and quality of life. Eosinophil count decreased, reflecting a reduced airway inflammation. Both FEV1 and % FEV1 showed significantly higher values, alongside improvements in FeNO. Furthermore, exacerbation frequency declined, accompanied by a reduction in the proportion of patients requiring continuous OCS therapy. EGPA subgroups did not substantially differ from the other two (Table 4). Asthma control and sinonasal symptoms both ameliorated, as shown by ACT and SNOT22 scores. Blood eosinophil counts declined markedly, confirming reduced inflammation. FEV1 and % FEV1 highlighted enhanced pulmonary function. FeNO levels showed a favorable trend. Notably, OCS use decreased substantially, underscoring the steroid-sparing effect of mepolizumab in this population.

**Figure 1:**
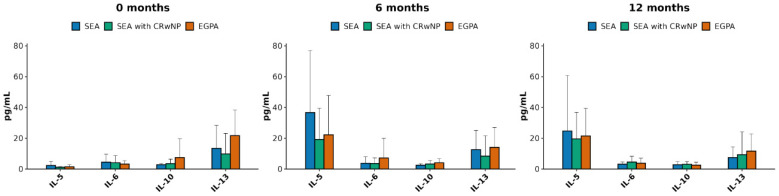
Cytokine profiles across the different experimental groups at the time points investigated: left panel T = baseline; middle panel T = 6 months; right panel T = 12 months. Severe eosinophilic asthma (SEA), chronic rhinosinusitis with nasal polyps (CRwNP), and eosinophilic granulomatosis with polyangiitis (EGPA).

**Figure 2:**
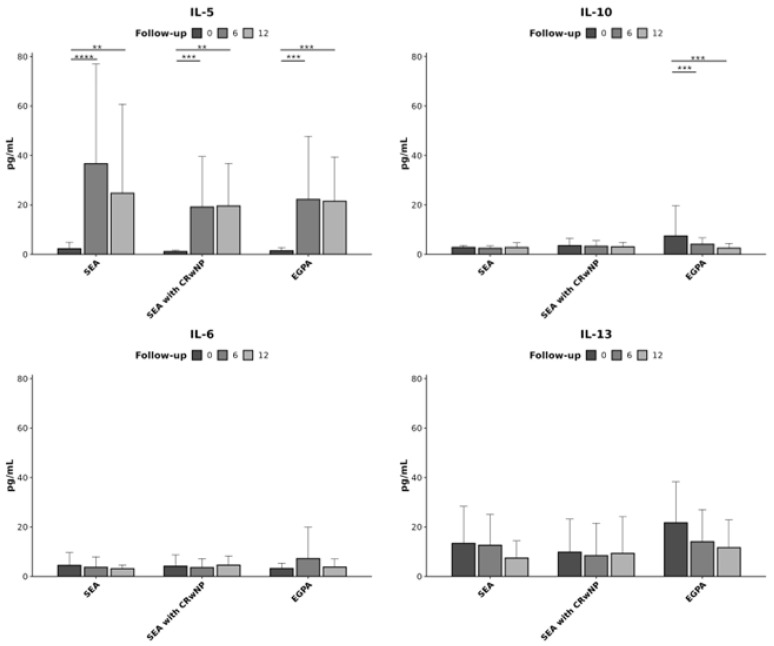
Comparative cytokine analysis where aach panel describes a single cytokine in the different disease groups at the different time points. Severe eosinophilic asthma (SEA), chronic rhinosinusitis with nasal polyps (CRwNP), and eosinophilic granulomatosis with polyangiitis (EGPA). Statistically significant differences are indicated by * (** for *p* ≤ 0.01, *** for *p* ≤ 0.001, **** for *p* ≤ 0.0001).

Cytokines profiles at baseline and follow up are described in Figure 1 and Figure 2. When comparing the three study population subgroups (Figure 1) both T2 and non-T2 cytokines were expressed, without statistically significant differences according to the diagnosis or the timepoint. At baseline (Figure 1, left panel), IL-5 and IL 6 were slightly more expressed in SEA patients; IL-13 and IL-10, conversely were higher in EGPA subgroup. At six months (Figure 1, middle panel), IL-5, IL-10 and IL-13 demonstrated a similar—although more marked—expression profile compared to baseline, whilst IL-6 concentration on the opposite became higher in EGPA patients. After 12 months from mepolizumab initiation (Figure 1, right panel), IL-5 concentrations decreased in all groups compared to the six months assessment. IL-6 and IL-13 levels did not show variations, and IL-10 concentrations decreased constantly in EGPA patients when compared to the previous timepoints and with respect to the other groups.

When analyzing the cytokine concentrations by diagnosis subgroups at different time points (Figure 2) significant changes could be observed for IL-5 and IL-10.

Supplementary Figure S1 summarizes the analysis of the correlation among clinical scores, biomarkers and cytokines levels (using Spearman’s rank correlation coefficient). At baseline an indirect correlation between ACT and IL-6, FEV1 and IL-6, FEV% and IL-6 was observed in SEA patients. Furthermore, there were no correlations after six months, while at 12 months an inverse correlation was shown between ACT and IL-6. Finally, a positive correlation between ACT and IL-5 was observed.

In patients with SEA with CRwNP, an indirect correlation between FEV1 and FEV1% with IL-6 was shown at baseline. A positive correlation was observed for IL-6 and ACT at six months. At 12 months a positive correlation could be described between FEV1 and IL-5.

Moreover, in the EGPA group, the correlation analysis between clinical scores and cytokines levels, at the baseline, showed a positive trend between eosinophils and IL-5. Conversely, a negative correlation was detected for OCS and IL-5. Finally, at 12 months a positive correlation was shown between ACT and IL-10, as well as OCS and IL-6.

## 4. Discussion

Our real-life observational prospective study investigated the trajectory of the molecular inflammatory profile in terms of T2 and non-T2 cytokines in patients affected by severe eosinophilic asthma with/without CRwNP or previous EGPA and prescribed mepolizumab 100 mg/four weeks over a one-year follow-up.

In terms of clinical outcomes, our observations proved the efficacy of mepolizumab 100 mg in different SEA patients regardless of coexisting determinants of potential higher disease burden, including CRwNP and previous EGPA history, and despite different clinical severity grade at baseline, confirming what registration and clinical trials had separately found for the different clinical indications for mepolizumzb [15,24].

Mepolizumab markedly reduced blood eosinophil counts in patients with SAE, consistent with its mechanism of action and therapeutic objectives. The same results were seen in asthma patients affected by CRwNP and with a history of EGPA. It has been previously described that the decrease in blood eosinophil count associates with clinical scores, which also underlines the pivotal role of eosinophils in these diseases [25].

In terms of pulmonary function, the improvements documented in our cohort are in line with the existing literature demonstrating that mepolizumab enhances pre-bronchodilator FEV1 compared to placebo. This effect was particularly pronounced in patients with concomitant nasal polyps, who exhibited greater FEV1 gains than those without nasal polyps, paralleling the significant increase observed in our asthma with CRwNP subgroup. By contrast, the SEA group showed a modest but non-significant rise in FEV1, in agreement with results from the SIRIUS trial [26].

These observations gained further relevance when considered alongside the marked steroid-sparing effect, which is a crucial benefit in the management of severe asthma and EGPA given the numerous OCS related side effects [14,27]. In our cohort, a substantial reduction in OCS dependence was observed, particularly within the EGPA subgroup.

Comparative evaluation of T2 and non-T2 molecular profile in SEA patients with different clinical expressions represents the novelty of our work. Of note, the normal cytokines levels in healthy subjects are not yet known or at least they are not standardized, neither in the clinical setting nor in the literature.

When comparing T2 and non-T2 cytokines in the three considered patients’ subgroups, the baseline assessment reveals the expression of all investigated cytokines in the three subgroups, suggesting a concomitant ongoing T2 and non-T2 inflammatory process. The latter being more apparent in IL-10 levels for EGPA patients. These findings are consistent with a higher degree of systemic involvement that characterizes this condition, which is in the remission phase except from the airway manifestations.

Our data on the IL-5 trend in asthmatic patients demonstrate an increase in IL-5 expression at the six months follow-up, which, although clinically silent, are different from what was observed in a recent paper by Palacionyte and colleagues [28], where IL-5 concentrations decreased over time. These contrasting results may be due to the different investigated patients’ population and the different study design and clinical criteria, as well as the background therapy at the time of enrolment. Furthermore, the pharmacokinetic of mepolizumab itself may account for our findings In fact, it is possible that our measurements included both free IL-5 and IL-5/mepolizumab complex. An anti-mepolizumab antibody could be implemented in our study panel in the future to further investigate the IL-5 concentration increase over time and the half-life of mepolizumab/IL-5 complex, as suggested by the literature [29].

In addition, the progressive OCS tapering might also contribute to the IL-5 increase due to the known steroid-sensitivity of eosinophilic inflammation. In fact, at baseline, a considerable proportion of patients, almost 90% for the EGPA subgroup and 22% of patients in the other subgroups, were receiving OCS therapy, whilst at 12 months, only 21% of patients from the EGPA subgroup, 7.7% of the SEA with CRwNP patients, and 5% of the SEA patients were still taking OCS.

However, we also observed in our population a decrease at 12 months after the peak concentration that was reached at six months. IL-13 is another key cytokine in type 2 inflammation. It promotes eosinophilic inflammation, mucus overproduction and epithelial dysfunction, which are common pathophysiological features of asthma and CRwNP. The decreasing trend in IL-13 could be related to the decrease in blood eosinophils number and IL-5: it has been proven in experimental models that IL-13 triggers eotaxin production and the inflammatory asthmatic effect of IL-13 levels is dependent on its ability to induce eotaxin expression and increase blood eosinophils count. Therefore, the decrease trend of IL-13 shown by our data could be related to the effects of Mepolizumab on IL-5 and thus on IL-13 [30,31], in agreement with previous reports.

IL-6 plays a central role in general inflammation. It can amplify both acute and chronic inflammation; it can also promote inflammation by inducing the liver to produce acute-phase proteins like C-reactive protein (CRP). It also promotes the differentiation of B cells to plasma cells and the polarization of T cells to Th17 cells. Nevertheless, its role in asthma is not yet clear and its role as a biomarker and as therapeutic target has been explored without any reliable result [32].

IL-6 also seems to contribute to nasal polyposis chronic inflammation but its precise function in this setting is not yet fully elucidated [33]. The same happens in EGPA where there are not enough data supporting the role of IL-6 in this disease. Our results showed a decreasing trend for IL-6 expression in asthma and EGPA subgroups, which is consistent with the mepolizumab modulatory effect on inflammation. The non-statistically significant decrease in IL-6 serum concentration over time in patients with chronic rhinosinusitis with nasal polyps is difficult to explain but is likely due to patients’ variability.

IL-10 is a cytokine that primarily acts as an anti-inflammatory agent to prevent excessive inflammatory tissue damage. It suppresses the production of pro-inflammatory cytokines, down-regulates the activity of macrophages and other immune cells, promoting immune tolerance. IL-10 is secreted by macrophages, T cells (regulatory T cells and CD4+ T cells), B cells, and dendritic cells which are considered among the major producers. Other IL-10 producers are epithelial cells, keratinocytes, NK cells, neutrophils, and mast cells. IL-10 can down-regulate cytokine production from Th2 cells as well as Th1 cells. Patients with asthma have a relatively reduced ability to produce IL-10 and low concentrations of the cytokine usually correlate with asthma development and severity. However, IL-10 cannot be defined as a biomarker for asthma severity [34]. Increased IL-10 expression in CRwNP has already been reported. Together with the increased production of the inflammatory cytokines like IL-5, IL-17A, IL-25 and IFN-γ, it seems that IL-10 contributes to the pathogenesis and eventual evolution of CRwNP [35]. In addition, the role of IL-10 in EGPA is not yet clear. Our data show stable serum concentrations of IL-10 in patients affected by asthma and CRwNP over time, while there is a statistically significant decrease in the concentration of IL-10 in patients affected by EGPA every six months. The decrease in EGPA patients IL-10 levels could be related to OCS discontinuation as, according to the literature, OCS use can increase IL-10 production in the above-mentioned cells [36,37]. Moreover, the decrease in IL-10 levels could be due to the effect of mepolizumab on the TH2 inflammation and eosinophil activation. With less inflammatory stimuli, the immune system has a lower need of IL-10, because the feedback loop is no longer strongly activated. The direct correlation between single cytokines expression and specific clinical parameters or common biomarkers has been rarely explored, some data suggesting a parallelism between cytokines levels and blood eosinophils/FeNO values in asthma patients [28]. We observed from preliminary findings that a higher level of IL-6 can be detected in SEA and SEA with CRwNP patients with more compromised asthma control, which is coherent with the hypothesis that non-T2 cytokines might contribute to inflammatory cascade in asthma, as discussed above. The positive correlation between IL-5 expression and asthma related clinical variables (ACT, FEV1) observed in SEA and SEA with CRwNP patients is more difficult to explain. However, the same methodological issues mentioned above potentially explaining the increasing IL-5 trend over the biologic treatment course might provide an interpretation.

The systemic evaluation of cytokines in the investigated patient population highlighted the importance of molecular characterization in the context of asthma, enabling a deeper understanding of the different experimental groups. Moreover, our work illustrated, as a point of novelty, the effect of mepolizumab treatment on the anti-inflammatory regulator IL-10, more marked in EGPA patients, which was, up to date, never investigated in this setting. It is in fact intriguing to consider the effect of biologicals not only on target downstream effectors of type 2 inflammation but also on other opposite molecular actors such as IL-10.

Taken together, our observations confirm the ability of mepolizumab to modulate non-T2 immune pathways and highlight the persistence of slightly different molecular profiles in different severe asthma patients depending on concomitant conditions, which is relevant for the long-term follow-up and potential association therapy combining options that address different targets.

The small sample size and the lack of randomization represent limitations which can potentially affect the validity of our findings. However, as an element of novelty, the profile and trajectories of both T2 and non-T2 cytokines, especially anti-inflammatory regulators such as IL-10, have been explored in severe eosinophilic asthma patients, highlighting the importance of molecular biomarkers of inflammation besides eosinophils when investigating complex patients.

More research in the field is required to further investigate and understand the role of serum cytokines related to inflammatory mechanisms in eosinophil-driven conditions.

## Figures and Tables

**Table 1:** Descriptive statistics of the population at baseline according to subgroups. * *p* < 0.05.

	SEA	SEA with CRwNP	EGPA
	(*n* = 18)	(*n* = 9)	(*n* = 18)
AGE (years)	58.72 ± 14.56 *	62.89 ± 12.64 *	60.33 ± 19.95 *
SEX	F = 13 (72%)	F = 4 (44%)	F = 10 (55%)
	M = 5 (28%)	M = 5 (56%)	M = 8 (45%)
BMI (Kg/m^2^)	25.24 ± 4.91 *	25.41 ± 3.52 *	24.59 ± 4.71 *
ACT	15.13 [8–22] *	12.63 [7–25] *	19.63 [10–25] *
SNOT22	-	42.11 [10–67] *	25.4 [0–49] *
EOSINOPHILS (eos/μL)	951.18 ± 865.10 *	570.00 ± 370.64 *	1200 ± 1793.71 *
FEV1 (L)	2.22 ± 0.80 *	2.08 ± 0.74 *	2.61 ± 1.14 *
% FEV1	78.89 ± 20.73 *	71.78 ± 14.45 *	89.5 ± 23.75 *
FeNO (ppb)	50.11 ± 53.45 *	74.83 ± 60.54	69.49 ± 37.45 *
OCS CYCLES/YEAR (mean)	5.50 [0–18]	3 [0–12]	0 [0–0]
% CONTINUOUS OCS	22.22% (4)	16.66% (1.5)	89.47% (16)

**Table 2:** Trends of clinical characteristics over the study period in SEA patients. * *p* < 0.05.

SEA (*n* = 18)			
	0 Months	6 Months	12 Months
ACT	15.13 [8–22] *	22.18 [13–25] *	23.25 [16–25] *
EOSINOPHILS (eos/μL)	951.18 ± 865.10 *	67.00 ± 58.96 *	72.86 ± 65.76 *
FEV1	2.22 ± 0.80 *	2.35 ± 0.9 *	2.65 ± 0.59 *
% FEV1	78.89 ± 20.73 *	89.13 ± 20.01 *	86.68 ± 32.57 *
FeNO (ppb)	50.11 ± 53.45 *	35.50 ± 34.23	18.16 ± 6.87 *
OCS CYCLES/YEAR	5.50 [0–18]	0 [0–1]	0 [0–3]
% CONTINUOUS OCS	22.22%	5.26%	5%

**Table 3:** Trends of clinical parameters over the study time frame in SEA with CRwNP patients. * *p* < 0.05.

SEA with CRwNP (*n* = 9)			
	0 Months	6 Months	12 Months
ACT	12.63 [7–25] *	20.75 [15–25] *	19.78 [0–25]
SNOT22	42.11 [10–67] *	26.29 [6–43] *	20.33 [16–25] *
EOSINOPHILS (eos/μL)	570.00 ± 370.64 *	12.67 ± 15.53	65.00 ± 41.23
FEV1	2.08 ± 0.74 *	2.43 ± 0.88 *	2.57 ± 0.88 *
% FEV1	71.78 ± 14.45 *	75.76 ± 33.90 *	83.29 ± 11.21 *
FeNO (ppb)	74.83 ± 60.54	24.48 ± 13.59 *	24.57 ± 8.60 *
OCS CYCLES/YEAR	3 [0–12]	0 [0–0]	0 [0–1]
% CONTINUOUS OCS	16.66%	7.69%	7.69%

**Table 4:** Trends of clinical parameters over the study period in EGPA patients. * *p* < 0.05.

EGPA (*n* = 18)			
	0 Months	6 Months	12 Months
ACT	19.63 [10–25] *	23.42 [20–25] *	23.50 [20–25] *
SNOT22	25.40 [0–49] *	12.20 [0–30] *	11.90 [1–24] *
EOSINOPHILS (eos/μL)	1200 ± 1793.71	110 ± 199.72 *	62.50 ± 51.06 *
FEV1	2.61 ± 1.14 *	2.91 ± 1.04 *	2.68 ± 0.74 *
% FEV1	89.50 ± 23.75 *	98.44 ± 19.97 *	99.60 ± 17.32 *
FeNO (ppb)	69.49 ± 37.45 *	33.62 ± 28.18 *	27.43 ± 22.05 *
OCS CYCLES/YEAR	0 [0–0]	0 [0–0]	0 [0–1]
% CONTINUOUS OCS	89.47%	57.89%	21.05%

## Data Availability

Data are available upon request.

## Supplementary Materials

The following supporting information can be downloaded at: https://www.mdpi.com/article/10.3390/biomedicines14030541/s1, Supplementary Figure S1: Correlation analysis between cytokine concentrations and clinical parameters at different time points in the different experimental groups.

## Abbreviations

The following abbreviations are used in this manuscript:

ACR	American College of Rheumatology
ACT	Asthma Control Test
ANCA	Anti-Neutrophil Cytoplasmic Antibodies
ATS	American Thoracic Society
BVAS	Birmingham Vasculitis Activity Score
CRwNP	Chronic rhinosinusitis with nasal polyps
CRSsNP	Chronic rhinosinusitis without nasal polyps
Ig	Immunoglobuline
INCS	Intranasal corticosteroids
ERS	European Respiratory Society
ESS	Endoscopic sinus surgery
EGPA	Eosinophilic granulomatosis with poliangiitis
EULAR	European Alliance of Associations for Rheumatology
FEV1	Forced Expiratory Volume in 1 Second
IL	Interleukin
OCS	Oral corticosteroid
ppb	Parts per billion
RCT	Randomized control trial
SEA	Severe eosinophilic asthma
