# Supplementary material for: Trends of T2 and Non-T2 Cytokines During Mepolizumab Treatment in Different Asthma Patient Subgroups
Source: Biomedicines. 2026 Feb 27;14(3):541. doi: 10.3390/biomedicines14030541 (PMC13024023; doi:10.3390/biomedicines14030541)
Supplement: Supplementary file 1 [file biomedicines-14-00541-s001.zip › biomedicines-4103157-supplementary.pdf]

Supplementary Material.

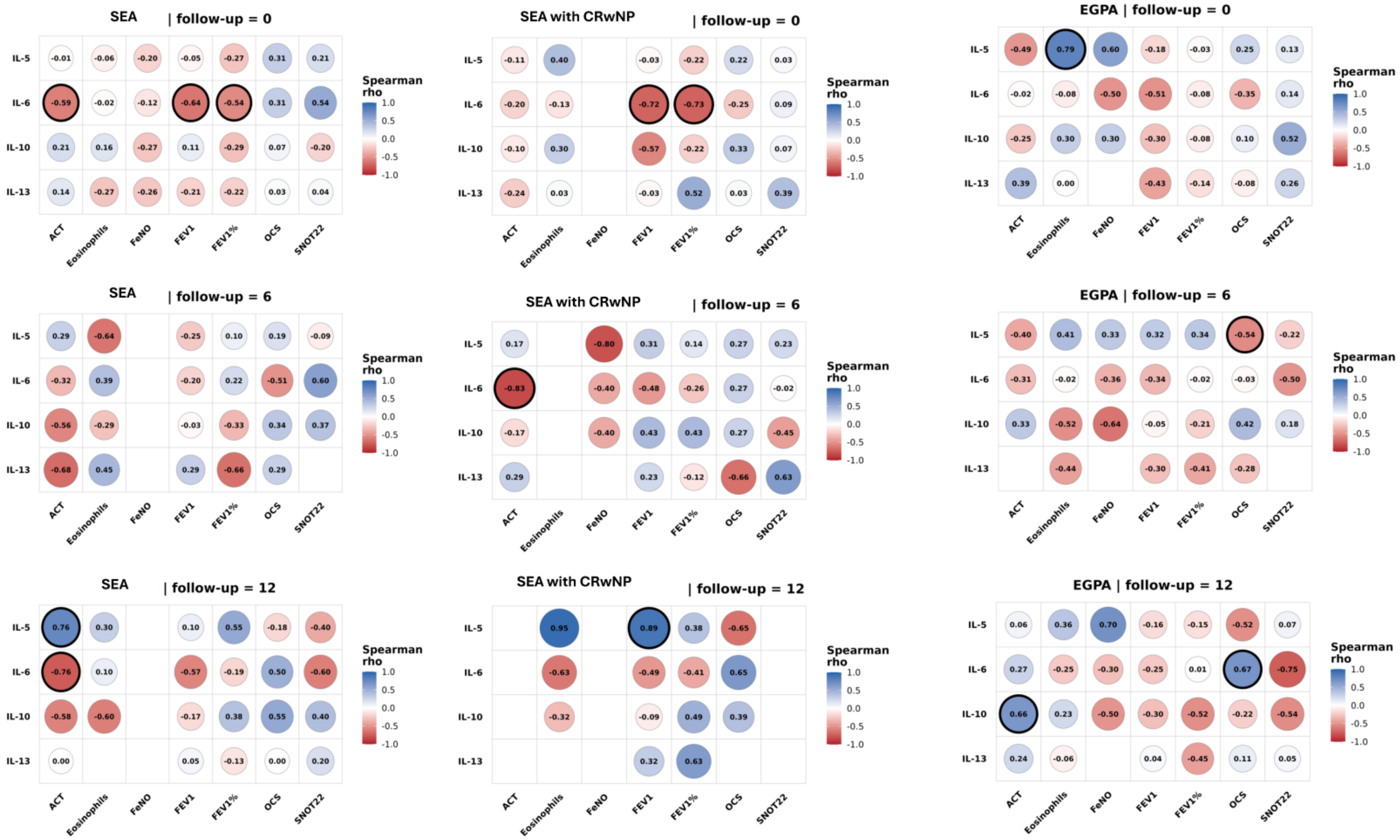

Supplementary Figure S1. Correlation analysis between cytokine concentrations and clinical parameters at different time points in the different experimental groups. The left column shows results for Severe Eosinophilic Asthma (SEA); the middle column shows results for Severe Eosinophilic Asthma with chronic rhinosinusitis with nasal polyps (SEA with CRwNP); and the right column shows results for Eosinophilic granulomatosis with polyangiitis (EGPA). Significant correlations are indicated by bold circles.
